# Two Cases of Cystic Fibrosis with Compound Heterozygous Variants Reported for the First Time

**DOI:** 10.4274/balkanmedj.galenos.2020.2019.11.128

**Published:** 2020-08-11

**Authors:** Sinem Yalçıntepe, Hakan Gürkan, Engin Atlı, Niyazi Cenk Sayın, Ümit Nusret Başaran

**Affiliations:** 1 Department of Medical Genetics, Trakya University School of Medicine, Edirne, Turkey; 2 Department of Perinatology, Trakya University School of Medicine, Edirne, Turkey; 3 Department of Child Surgery, Trakya University School of Medicine, Edirne, Turkey

To the Editor,

Cystic fibrosis (CF) is the most common and autosomal recessive genetic disease in the population. It’s frequency is observed to be 1 in 2,500 live births globally. In Turkey, its incidence is 1 in 3,000, despite the limited number of reported studies. It is believed that this rate is higher due to frequent consanguineous marriages. The *CFTR* gene is on the seventh chromosome. It is 230-kb long, encodes 1,480 amino acids, and contains 27 exons. The product protein of *CFTR* gene, which is known as protein-regulating CF transmembrane regulatory (CFTR), basically acts as a chlorine channel. To date, about 1000 mutations have been reported in the clinical and genetically heterogeneous CF (1). In this letter, we aimed to report two CF cases with compound heterozygous CFTR variants, which are determined for the first time.

Case 1; a one-month-old female child was referred to our clinic after the suspicion of CF in the neonatal screening result. She is the first live-born child of the non-consanguineous couple. She did not show any problems after section-cesarean birth at term. There was no pathologic history or a history of CF in the parents, except for Rh incompatibility. The birth weight of the child was 4,050 grams (>90 p), and the birth length was 52 cm (75-90 p). There was no anomaly observed in the patient after a physical examination. After obtaining the written informed consent form, complete sequencing of the CFTR gene by next-generation sequencing revealed that two different variants in trans, NM_000492.4(CFTR):c.3909C>G (p.Asn1303Lys) was inherited from the mother, whereas NM_000492.4(CFTR):c.2657+5G>A variant was inherited from the father (Figure 1). These variations have been previously reported in patients with CF and/or carriers of CF, but not reported together as compound heterozygosity in a patient. Our patient was diagnosed with CF after this result. Additionally, the family was provided with genetic counseling. In our knowledge, this case is the first for this result of compound heterozygosity.

Case 2; a 29-year-old primigravida woman with fetal intestinal dilatation and hyperechogenic intestines at 26 weeks’ gestation from the gynecology and obstetrics clinic was referred for genetic counseling to our medical genetics clinic. The prenatal ultrasonography revealed a 26-week-old singleton fetus. Dilated, thickened, and concentric (snail sign) intestinal loops indicated intestinal volvulus. After prenatal genetic counseling, the family did not accept prenatal diagnosis. We analyzed the parents for CF. The mother had heterozygous NM_000492.3(CFTR):c.1624 G>T (p.Gly542Ter) variation, whereas the father had heterozygous NM_000492.3(CFTR):c.489+1 G>T variation (Figure 3). Because of the premature rupture of membranes and fetal distress, the fetus was delivered by caesarean section at 36 weeks’ gestation. The birth weight was 3,190 (25^th^ centile) grams, and the birth length was 45 cm (3^rd^ centile). She immediately underwent surgery after birth due to meconium ileus (Figures 2, 3). In the surgery, the surgeons resected the atretic segment that involved the proximal 6 cm of the ileum and performed end-to-end anastomosis. She was monitored in intensive care unit for 45 days. Thereafter, she was taken to gastroenterology clinic for insufficient weight gain. The family provided the written informed consent form. In our medical genetics clinic, we analyzed the case for variations in the CFTR gene, compound heterozygosity for NM_000492.3(CFTR):c.489+1 G>T and NM_000492.3(CFTR):c.1624G>T (p.Gly542Ter). As a result, we identified pathogenic variations in these cases. Both mutations have previously been described separately in patients with CF; however, with this result, this infant is the first reported case in the literature.

CFTR is primarily located in the apical membranes of epithelial cells and acts as a chlorine channel in the cells. Thus, water and salt transport from the cell membrane is affected in CF, further resulting in changes in the composition of the fluid secreted in the airways, pancreas, gastrointestinal tract, sweat glands, and other exocrine tissues. These changes cause the infection agents to settle easily, with increased viscoelasticity of the mucus in the lungs and saltier epithelial gland fluid. Because CF is a disease involving more than one system, it manifests with many different clinical signs and symptoms (2).

In our two cases, we detected different compound heterozygous mutations in the *CFTR* gene with the help of genetic analysis. Furthermore, autosomal recessive inheritance results in the diagnosis of compound heterozygosity. Recent years have witnessed innovations in the treatment of the disease. Moreover, studies on drugs that will activate the mutant *CFTR* gene are still under investigation (3,4). As the genetic characteristics of the disease are elucidated, gene therapy will be possible in the future.

## Figures and Tables

**Figure 1:**
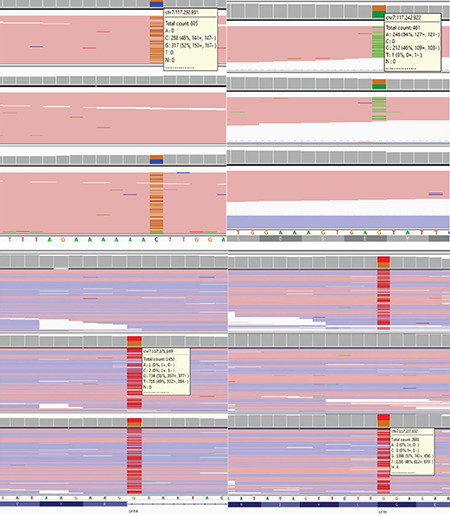
Molecular screenings of *CFTR* gene in cases 1 and 2. Both the cases show compound heterozygosity for *CFTR* gene mutations, in which the parents are the carrier of the variations.

**Figure 2:**
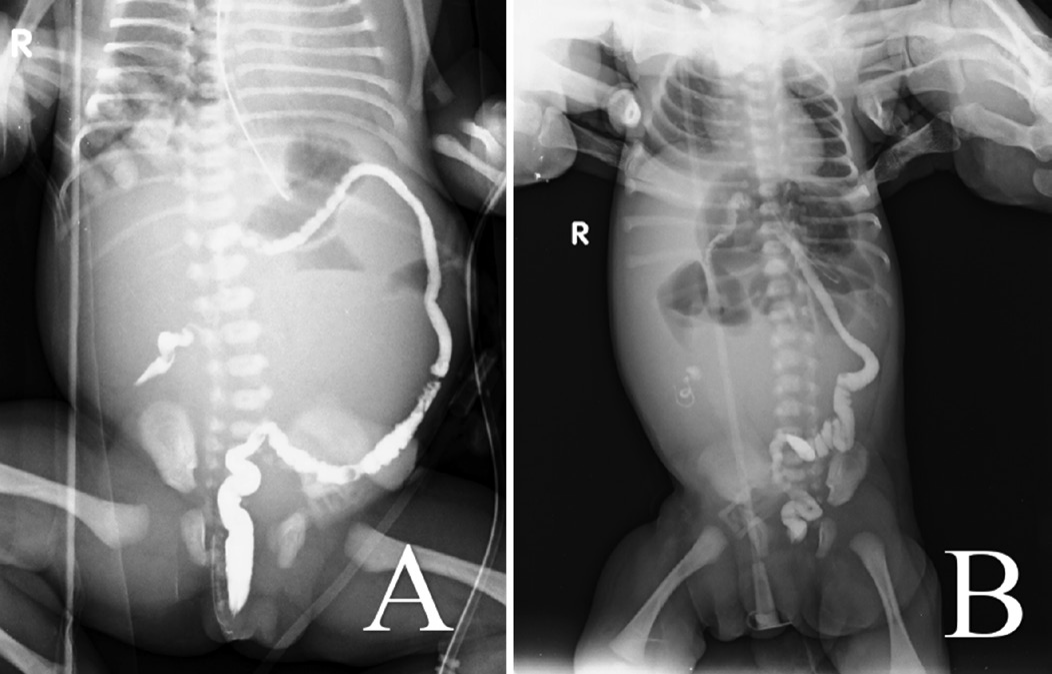
A. Pre-operative and B. Post-operative abdominal radiography of case 2. Pre-operative screening shows the blockage of ileus due to meconium ileus, whereas post-operative screening shows that there is no blockage after surgery.

**Figure 3:**
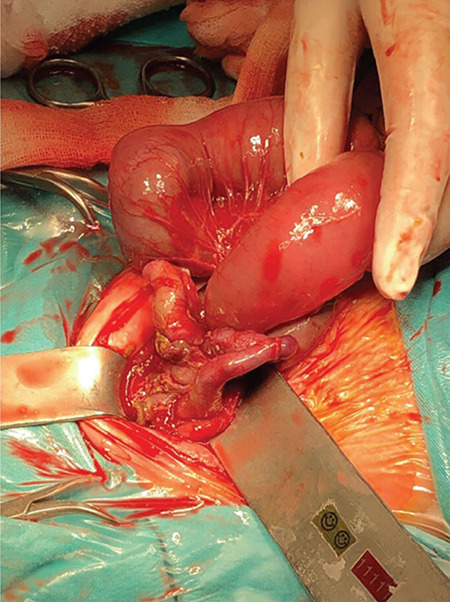
Surgical image of case 2 with meconium ileus. The figure shows the enlargement of bowel due to blockage of ileus with meconium.
